# Does an early home-based progressive resistance training program improve function following total hip replacement? Results of a randomized controlled study

**DOI:** 10.1186/s12891-016-1023-x

**Published:** 2016-04-21

**Authors:** Tosan Okoro, Rhiannon Whitaker, Andrew Gardner, Peter Maddison, John G. Andrew, Andrew Lemmey

**Affiliations:** Department of Orthopaedics, Ysbyty Gwynedd, Betsi Cadwaladr University Health Board, Bangor, UK; College of Medicine, Swansea University, Swansea, SA2 8PP UK; North Wales Organisation for Randomised Trials in Health (NWORTH), Bangor University, Bangor, UK; School of Sport Health and Exercise Science, Bangor University, Bangor, UK

**Keywords:** Progressive resistance training, Home based rehabilitation, Total hip replacement

## Abstract

**Background:**

In-hospital progressive resistance training (PRT) has been shown to be an effective method of rehabilitation following hip surgery. The aim of this study was to assess whether a home-based PRT program would be beneficial in improving patients’ muscle strength and physical function compared to standard rehabilitation.

**Methods:**

Subjects (*n* = 49) either received home-based PRT rehabilitation (*n* = 25) or standard rehabilitation (*n* = 24) in a prospective single blinded randomized trial carried out over a two-year period. The primary outcome measure was the maximal voluntary contraction of the operated leg quadriceps (MVCOLQ) with secondary measures of outcome being the sit to stand score (ST), timed up and go (TUG), stair climb performance (SCP), the 6 min walk test (6MWT), and lean mass of the operated leg (LM).

**Results:**

Twenty-six patients completed follow up at 1 year (*n* = 13 per group) for the final comparative analysis. All the outcome measures showed marked progressive improvements from the baseline measures at 9–12 months post op (Estimated effect (std error); p value)- MVCOLQ 26.50 (8.71) N *p* = 0.001; ST 1.37 (0.33) *p* = 0.0001; TUG −1.44 (0.45) s *p* =0.0001; SCP −3.41(0.80)s *p* = 0.0001; 6MWT 45.61 (6.10)m *p* = 0.0001; LM 20 (204)g *p* = 0.326) following surgery for both groups. Overall, there was no significant effect for participation in the exercise regime compared with standard care for all outcomes assessed.

**Conclusions:**

Overall, this study demonstrated that there is no significant difference between the two groups for participation in the home-based PRT exercise programme when compared to standard care for all outcomes.

**Trial registration:**

ISRCTN 1309951. Registered February 2011.

**Electronic supplementary material:**

The online version of this article (doi:10.1186/s12891-016-1023-x) contains supplementary material, which is available to authorized users.

## Background

Centre-based progressive resistance training (PRT) regimes for post- total hip replacement (THR) patients have been shown to improve objective measures of physical performance (e.g. 30 % higher sit to stand score, 30 % higher gait speed and 28 % higher stair climb performance [1]), but unfortunately require patients to exercise under supervision making program delivery expensive [2].

Addressing these issues has led to the assessment of home-based rehabilitation programs; also shown to be effective in improving function post-THR. However, at the time the current study was commenced, the two home-based interventions available in the literature featured programs initiated between 4 and 48 months following THR, with neither assessing the retention of benefits at follow up [3, 4]. Jan et al. [3] demonstrated improvement in the hip muscle strength of the operated side (~20 %), as well as improvement in walking speed (~24 %) after a 12-week program commenced between 18 and 48 months following surgery. Similarly, Trudelle-Jackson and Smith [4] showed an improvement in hip flexor and extensor strength (41 and 48 % respectively) for patients undergoing an exercise intervention compared to standard regimes after an 8 week program that included PRT, with the intervention commenced at least 4 months post-THR. A possible alternative to the centre-based programmes would be a home-based rehabilitation program that features PRT, and commences in the immediate post-surgical period with longer follow up. Thus the aim of this study was to perform a pilot study as proof of concept, assessing whether an inexpensive home-based PRT program with weekly supervision in the early post-operative phase after total hip replacement surgery was beneficial in improving muscle strength and physical function relative to standard rehabilitation at up to 1 year follow up.

## Methods

This was a prospective single blinded randomized trial carried out from April 2010 to March 2012. Patients undergoing elective THR surgery for osteoarthritis were recruited after local NHS Research Ethics (North West Wales) Committee approval (Ref 09/WNo01/52), and trial registration (ISRCTN13019951; registered February 2011). All participating patients gave their informed consent.

**Fig. 1:**
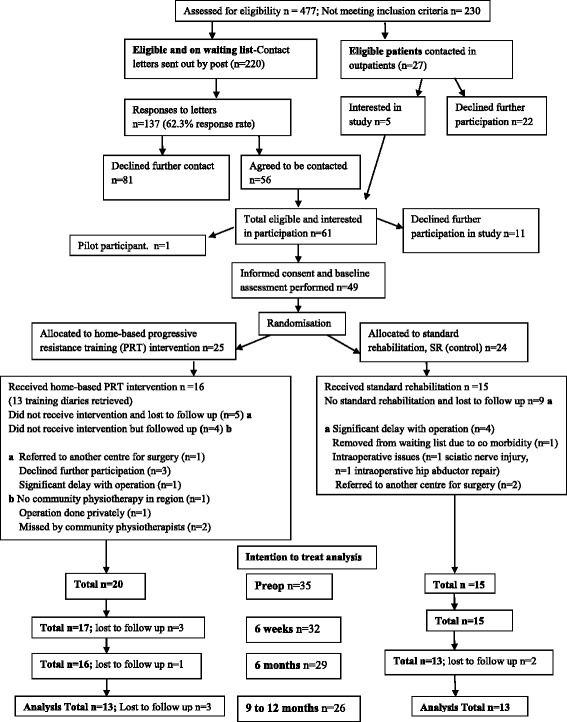
CONSORT flowchart for a 6 week home based progressive resistance training intervention study following total hip replacement surgery

Patients considered for this study were on the inpatient waiting list for THR at Ysbyty Gwynedd Hospital, Bangor, UK. They were eligible for participation if they had unilateral hip osteoarthritis requiring THR via a posterior approach with a 26 mm, 28 mm, or 32 mm femoral head, with the joint affected being the only severely arthritic joint, and no evidence of inflammatory arthropathy. The exclusion criteria were dementia, neurological impairment, cancer or other muscle wasting illness, unstable chronic or terminal illness, or any co-morbid disease that contraindicated resistance training. A Consolidated Standards of Reporting Trials (CONSORT) diagram [5] for patients recruited into the study after informed consent is shown in Fig. 1. A single patient acted as a pilot for the exercise intervention before subsequent one to one sequential individual randomization with stratification for age and gender [6] was performed for the other 49 study recruits. An offsite researcher performed randomization with the subsequent results only made available to physiotherapists in contact with the patients in the immediate post-operative period, with the assessor (TO) blinded to the results of randomization till the end of the study. A total of 25 patients were randomized to the home-based PRT group with 24 randomized to the standard rehabilitation (SR, control) group.

### Outcome measures

The primary outcome measure for this study was the maximal voluntary contraction (MVC) of the operated leg quadriceps (MVCOLQ; in Newtons (N)) The secondary outcome measures were the sit to stand score in 30 s (ST), other objective measures of function such as: timed up and go (TUG), stair climb performance (SCP) and the 6 min walk test (6MWT), as well as the lean mass of the operated leg as assessed by dual energy X-ray absorptiometry (DEXA) scanning. Assessments of the primary and secondary outcome measures were performed preoperatively, and at 6 weeks, 6 months and 9–12 months post-operatively by the first author (TO). All data was collected at the laboratories of the School of Sports, Health and Exercise Science, at Bangor University, Bangor, UK. The lean mass of the operated leg was assessed at 6 weeks and 9–12 months post-operatively.

### The outcome measures are described below

#### Maximal voluntary contraction of the operated leg quadriceps (MVCOLQ; in Newtons (N))

This primary outcome measurement was made using a handheld isokinetic dynamometer (CSD300, Chatillon-Ametek, Largo, FL, USA), which has been shown to have high test/retest reliability (0.97, *p* < 0.001; [7]). For the assessment, subjects sat on a medical table with arms across their chest. The curved push attachment of the dynamometer was positioned over the tibia just proximal to the 2 malleoli, and the subjects were instructed to attempt to straighten the leg forcefully. Following 2 sub-maximal familiarization trials, subjects were asked to exert force maximally for about 5 s on 3 further occasions. Between all 5 trials, a 1-min rest was observed. Peak force produced during each of the 3 maximal trials was recorded with the best score noted.

#### Sit to stand in 30 s (ST) score

This is the maximal number of times the subject was able to rise, with arms crossed over their chest, from a standardized chair (seat height 43 cm) in 30 s, and is a test designed to reflect the ability to perform activities of daily living (ADLs; [8]). A moderately high correlation exists between ST performance and maximum weight-adjusted leg-press performance for both men and women (*r* = 0.78 and 0.71, respectively) supporting the criterion-related validity of the sit to stand test as a measure of lower body strength [8]. Construct (or discriminant) validity of the chair-stand has been demonstrated by the test’s ability to detect differences between various age and physical activity level groups [8]. This test has an intra class correlation coefficient of 0.80 [9].

#### Timed up and go (TUG) in seconds (s)

The time taken in seconds for subjects to rise from a standard armchair, walk at a safe and comfortable pace to a cone 8 ft away, and return to a sitting position (back against the chair). Test-retest reliability estimates of 0.75 (type 2, 1 intraclass correlation coefficient (ICC)) for patients awaiting hip or knee replacement surgery have been demonstrated.

#### Stair Climb performance (SCP)

The time taken to ascend 14 standard steps of 20 cm height each in a usual manner and at a comfortable pace. The SCP has test-retest reliability (ICC) of 0.90.

#### Six-minute walk test (6MWT)

The distance covered (metres) in a level corridor over a 6-min period. Originally conceived as an outcome measure for patients with respiratory problems. It has been shown to have high reproducibility in different patient populations. It has the advantage of being reflective of patients’ ability to perform activities of daily living. It has a test-retest reliability estimate (ICC) of 0.94.

#### Lean mass of the operated leg

Whole body DEXA was performed using a pencil-beam scanner (QDR1500, Hologic, Bedford, Massachusetts) to determine total and regional (left and right arm, left and right leg, trunk, head) lean fat and bone mass. The lean mass value in grams for the operated leg of the subjects assessed whether the home-based PRT intervention increased muscle mass in the involved leg compared to standard rehabilitation (SR; control). A calibration standard was scanned daily, and measurement accuracy was measured by scanning a water/oil phantom of known proportions (41 % fat) monthly. The coefficient of variation of repeated measurements using the DEXA is between 1–3 % [10].

After informed consent, baseline preoperative assessment, and subsequent THR, patients in the study were randomized to either a home-based PRT intervention or SR (control) for the immediate (6 week) post operative period. These interventions are described below:

### Prescribed home-based PRT exercise intervention

This was devised by convening a discussion group of hospital and community physiotherapists (*n* = 5; all with more than 5 years experience of treating patients following THR). For patients randomized to home-based PRT, the exercises to be performed at home were demonstrated to them as inpatients by the attending physiotherapist on post-operative day 2. On discharge home, a qualified physiotherapist saw them and initiated the PRT regime between post-operative days 4 to 7. The exercises performed were: sit to stand, block stepping, stair climbing, walking, sitting knee extension against resistance, and lateral weight transfer exercises. Ankle weights and foam blocks were used as inexpensive and adjustable forms of equipment to increase resistance for the knee extension and stepping exercises, respectively. Patients in the intervention group were instructed to perform a range of repetitions (0–3, 4–6, 7–10) depending on their initial physiotherapy assessment and then to progress, when able to, to achieve progressive overload, i.e. the addition of increased resistance over time (the decision to progress was reviewed and facilitated by weekly physiotherapy visits during each of the 6 exercise intervention weeks). Subjects were encouraged to exercise at least 5 times a week. The physiotherapists determined the progression subjectively based on the ability of the patient. This was a pragmatic trial and the attending physiotherapist did their best to assess the patients in terms of ability to enable progression to occur.

Training volume (multiplying the number of repetitions performed/day by the number of days) was monitored using a simple training diary with compliance assessed as a measure of practice ratio i.e. number of days the subjects actually carried out the program multiplied by the program duration in days (5 days a week for 6 weeks, i.e. 30 days).

### Standard rehabilitation, SR (control)

The SR (control) group received routine inpatient and/or outpatient physiotherapy as provided by the local physiotherapy service. The standard rehabilitation provided in this study typically involved home-based functional non-PRT exercises that was geared towards getting the patients safely mobile. These included weight bearing (performed against gravity) and functional (without external loading) exercises, as well as bed-based (e.g. buttock squeezes, leg sliding and straight leg raise)/bridging (targeting core abdominal muscles as well as lower back and hip)/postural exercises (focusing on strengthening muscles which have become overstretched and weak).

### Statistical analysis

Based on the assumption that the exercise intervention would lead to a 15 % increase in the muscle strength (MVCOLQ) of the home-based PRT group relative to the SR (control) group [1], with an alpha value of 0.05 and power of 0.8, it was determined that 10 experimental subjects and 10 controls would be needed to demonstrate a significant effect. The target of a total of 50 participants (25 per group) was set to allow for potential dropouts during the follow up period (9–12 months post-THR).

A mixed model repeated measures ANOVA was performed with the primary and secondary outcome measures as dependent variables. The null model to fit the grand mean for the outcome variables was run first, and then an unconditional model with no predictors was used to determine whether a model with varying intercepts was suitable as well as determining the variance in the outcome measures between subjects. After the addition of time-point indexing to assess whether the pattern of linear change over time varies, additional predictors (group randomization (fixed, between-subjects effects)) and the effect of the follow up time period (random, within-subjects effects) were added to the model to attempt to explain any overall change over time. An interaction term of randomization group and time was then added to the model and if this was not significant, it was removed from the final model applied. A p value <0.05 was considered statistically significant. SPSS version 18 (SPSS for Windows v18, Rel. 30.07.2009. Chicago: SPSS Inc) was used for all analysis.

## Results

Of a total of 49 patients recruited to this study, a total of 14 were lost to follow up preoperatively due to a variety of reasons; see CONSORT diagram (Fig. 1). Thirty-five patients were therefore included in the analysis (Demographic data for the eligible and recruited cohort (*n* = 49) is described in Table 1. Three patients were lost to follow up at each of the review time points with 26 patients completing 9–12 month final follow-up (final follow-up rate of 74.28 % (26/35)).

**Table 1:** Baseline demographic characteristics, and preoperative outcome measures for total hip replacement surgery rehabilitation trial participants

Characteristic/outcome measure	Home-based, progressive resistance training (PRT) group (*n* = 25)	Standard rehabilitation (control) group (*n* = 24)
Age in years (mean (SD))	65.15 (9.06)	66.33 (11.02)
Sex- Males (n)	10	14
Sex- Females (n)	15	10
Weight (kg)	78.88 (19.17)	81.46 (16.43)
Height (m)	1.67 (0.10)	1.66 (0.09)
BMI	28.04 (5.79)	29.44 (5.25)
Maximal Voluntary Contraction Operated Leg Quadriceps (N)	167.38 (77.04)	182.13 (73.05) *p* = 0.497
Sit to stand (ST) number performed in 30 s	8.92 (4.69)	8.20 (4.18) *p* = 0.574
Stair Climb Performance (SCP) in seconds (s)	14.70 (8.67)	18.13 (9.94) *p* = 0.204
Timed up and go test (TUG) in seconds (s)	13.35 (10.05)	12.06 (6.02) *p* = 0.589
Six Minute Walk Test (6MWT) in metres (m)	259.71 (116.57)	236.96 (108.69) *p* = 0.480

The values for the primary and secondary outcome variables preoperatively and at 9–12 month follow-up for the home-based PRT and SR (control) groups appear in Table 2. There were no statistically significant differences between the randomized groups preoperatively (Table 1).

**Table 2:** Absolute and change values (mean (SD)), and mixed model ANOVA results at final follow up for primary and secondary outcome measures for home-based progressive resistance training (PRT) and standard rehabilitation (control) groups preoperatively following total hip replacement surgery

	Preoperative^a^		9-12 months postoperatively		Change values (Difference between 9–12 months and baseline)		Mixed model ANOVA for change from baseline values effect (std. error)	
Primary Outcome	Home-based PRT *n* = 20	Standard rehabilitation (control) *n* = 15	Home-based PRT *n* = 13	Standard rehabilitation (control) *n* = 13	Home-based PRT *n* = 13	Standard rehabilitation (control) *n* = 13	Effect of Treatment SR > PRT	Effect of Time
MVCOLQ (N)	172.30 (85.10)	174.20 (70.30)	247.40 (85.10)	240.3 (87.4)	58.31 (95.43)	56.08 (61.66)	10.38 (23.72) *p* = 0.065	26.50 (8.71) *p* = 0.001^b^
Secondary Outcomes (Exploratory analysis)								
ST	9.30 (4.74)	8.26 (4.80)	13.21 (5.46)	14.16 (5.47)	3.64 (2.73)	4.75 (4.04)	1.43 (1.19) *p* = 0.239	1.37 (0.33) *p* = 0.0001^b^
Lean mass in grams (g) of the operated leg	8265 (2326)	7601 (1989)	8769 (2109)	7889 (2226)	200.15 (800.58)	194.08 (586.98)	280 (419) *p* = 0.508	20 (204) *p* = 0.326
TUG (s)	13.47 (11.06)	12.14 (6.90)	8.64 (3.23)	7.06 (1.31)	−3.74 (5.37)	−2.68 (2.35)	0.09 (2.64) *p* = 0.972	−1.44 (0.45) *p* = 0.0001
SCP (s)	13.74 (7.49)	17.80 (10.99)	8.32 (4.45)	7.64 (2.70)	−6.69 (5.08)	−7.71 (6.99)	−5.67 (2.61) *p* = 0.038^b^	−3.41 (0.80) *p* = 0.0001^b^
6MWT (m)	269.80 (115.0)	238.7 (110.5)	352.4 (109.3)	376.5 (49.9)	84.52 (52.41)	120.91 (88.59)	86.39 (27.94) *p* = 0.004^b^	45.61 (6.10) p = 0.0001

Key: *MVCOLQ* Maximal voluntary contraction of the operated leg quadriceps, *ST* Sit to stand, number of repetitions in 30 s, *TUG* Timed up and go test in seconds (s), *6MWT* Six minute walk test in metres (m), *SCP* Stair Climb performance in seconds (s)

^a^No statistically significant differences between groups

^b^Statistically significant

**Fig. 2:**
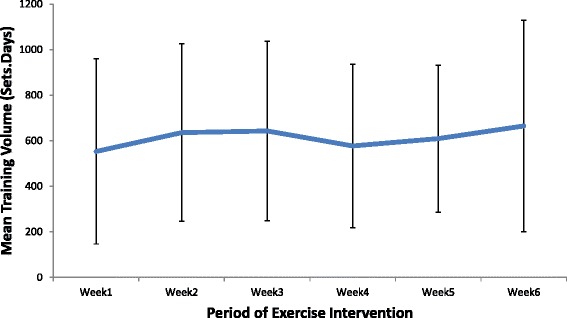
Mean Training Volume versus Period of Exercise Intervention in patients undertaking a home based progressive resistance training regime after total hip replacement

Thirteen patients who completed the home-based PRT exercise program returned exercise diaries (Fig. 1), with the average training volume over each of the 6 weeks shown in Fig. 2. There was a gradual increase in the calculated training volume from (mean (SD)) 583 (409) repetitions.days in week 1 to 687 (478) repetitions.days in week 6.

The average compliance to the prescribed program was 125 % (i.e. on average, the home-based PRT subjects completed 37.5 training days rather than the minimal requirement of 30 days), indicating that for the patients from whom training records were retrieved, the intervention was well tolerated.

An intention to treat analysis was performed (Fig. 1) and the mixed model repeated measures ANOVA output data for both the absolute values for the primary and secondary outcomes, as well as the change from baseline values for these measures, is incorporated into Table 2.

### Absolute values of the outcome measures

All the outcome measures (both primary and secondary) showed marked progressive improvements from the baseline measures in terms of absolute values following surgery for both groups. There was no effect of treatment, i.e. no differences between the home-based PRT or standard physiotherapy (control) groups, on the absolute values for any of the outcomes (MVCOLQ, sit to stand (ST) score, and lean mass of the operated leg) at any stage over the 9–12 month period of investigation.

### Changes in outcome variables from preoperative values

Improvement in 2 of the secondary outcome variables (SCP and 6MWT) at the 9–12 month post-surgery follow-up was observed for patients in the SR (control) group relative to the home-based PRT patients (Table 2).

### Effect of training volume on change in outcomes (dose response)

The training volumes (dose) were determined for the 13 study participants who completed exercise diaries. The only significant correlation identified was between volume and the change from baseline for the ST score, with an R-value of 0.639 (*p* = 0.019) at 6 weeks, 0.646 (*p* = 0.023) at 6 months, and 0.855 (0.002) at 9–12 months follow up. This indicates that higher training volume was associated with greater improvement in performance of the ST test, our surrogate measure of lower body function.

The median training volume was 4398 repetitions.days. Patients with higher values than this were classified as high training volume participants (HTVP, *n* = 7) whilst those with lower values were classified as low training volume participants (LTVP, *n* = 6). There was a significant effect at 9–12 months for being in the HTVP group compared to the LTVP for improvement in the ST test (mean (SD), 4.83 (2.04) increased repetitions vs. 1.50 (1.00), *p* = 0.010). There was also a significant effect at 9–12 months in the change from baseline values for the MVCOLQ, with the HTVP showing a mean improvement of 121 (84.63) Newtons (N) relative to a reduction of 5.33 (54.12) N in the LTVP (*p* = 0.034). There were no effects of training volume on the other primary and secondary outcome variables.

The compliance scores from the exercise diaries obtained combined with the analysis of training volume in the home-based PRT group indicate that the regime was well tolerated and in those patients who had high training volumes, significantly better improvements in two of the three principal outcomes were achieved and sustained for up to 9–12 months post-operatively.

## Discussion

This study shows that a home-based PRT program is just as efficacious as standard rehabilitation for improving quadriceps maximum voluntary contraction, sit to stand reps, skeletal muscle mass in the operated leg as well as timed up and go, in the year following total hip replacement surgery.

The SR (control) group showed greater improvement at final follow up in two objective measures of physical function, SCP and the 6MWT, relative to home-based PRT patients. All the measures assessed (except the lean mass of the operated leg) improved significantly over time for both treatment groups, which would be expected in this patient population as THR provides good pain relief and patients tend to become more physically active following surgery [11].

The home-based PRT intervention appears well tolerated, with the participants for whom exercise diaries were retrieved showing compliance rates on average of 125 % (i.e. 25 % more than the recommended minimum). There was a significant dose response for training, with significant differences observed between HTVP and LTVP in terms of the amount of improvement at 1 year in ST performance, and MVCOLQ. Compliance as a self-report measure is however a limitation to the study as it was impossible to accurately monitor how much the patients did in terms of the exercise prescription.

A study by Mikkelsen et al. [12]; published after this study was undertaken, also compared a home-based, intensified, early postoperative regime (12 weeks duration) after THR to standard rehabilitation. Consistent with our findings, they also found no differences between groups at their final follow up point (12 weeks). Again, like us, these investigators noted the expected improvement from baseline values in both groups following THR, and the prescribed resistance training regime was well accepted by patients on the basis of pain, compliance, and patient satisfaction [12]. The authors suggest that the lack of a significant benefit for the regime may be that participants’ additional training activities could not be controlled for. They also suggest that perhaps not all post-operative THR patients can perform exercises effectively without supervision [12].

Home-based interventions in the literature that have demonstrated a beneficial effect on restoration of muscle strength and objective function following THR have all been conducted some time after surgery i.e. 4 to 12 months [4] and at least 1.5 years [3]. Whilst the improvements in the objective measures of physical function assessed in these studies were significantly better in the exercise intervention groups than the controls (routine rehabilitation protocols), a significant level of impairment still persisted in these patients when final function was compared to a population of community dwelling age- and sex-matched adults without hip osteoarthritis.

The centre-based rehabilitation intervention conducted by Suetta et al. [1] was able to restore objective functional parameters such as “normal” gait speed (from 1.10 m/s (±0.50) to 1.43 m/s (±0.60)) following 12 weeks resistance training in patients immediately post-THR. As the follow up periods for the centre-based PRT studies in the literature do extend beyond the time frame of the interventions assessed, it remains to be seen whether the substantial functional improvements observed are maintained over a longer period.

For the 6MWT, the values obtained in our study after 9–12 months for the home-based PRT and SR (control) groups were 352 (±109) m and 377 (±50) m, respectively, which again is considerably lower than that for healthy community dwelling match adults without hip osteoarthritis (~527 m, [13]). This implies an average functional deficit in the present study population at final follow up of around 30 %; the same proportional deficit as for gait speed. Once again, this compares poorly to the improvement elicited by the centre-based rehabilitation intervention of Galea et al. [2] in which the values obtained after an 8 week PRT intervention was 427 m (an average deficit of 23 % from the normal value). These results suggest that centre-based regimes are able to produce better functional improvements.

There was a significant difference in the change from preoperative values at 12 months in 2 of the secondary outcome measures (SCP, 6MWT) in favour of the SR (control) group. This may be explained by the variability that exists in standard practice across the UK, with the regimes prescribed highly dependent on local resource allocation as well as physiotherapists’ preference. It demonstrates that a home-based PRT programme is just as effective but not better than pre-existing standard rehabilitation regimes.

The only home-based regimes in the literature that have improved functional outcome were performed between 6 months and 4 years after THR and were either for a short duration (8 weeks) with progressive resistance training [4] or for a long duration (12 weeks) [3]. The longer home-based higher intensity regime (12 weeks,(12)) performed on THR patients in the early post-operative period by Mikkelsen et al. [12] also provided no additional benefit to patients. The latter result in conjunction with ours appears to suggest that centre-based PRT regimes may be more effective in conferring a functional advantage in the early period following THR perhaps due to the additional supervision and the higher training intensity that is achievable. Additionally, the early period of surgical recovery (limb swelling, pain) may be more restrictive on patients in terms of performing training tasks effectively in the home setting. Undertaking an effective home-based intervention in this population may require the provision of trained home exercise specialists. This would ensure that patients under supervision to in the post-operative period might complete sufficiently intense regimes. This may only be effective in the post-recovery phase (>4 months) after THR and it may be appropriate to only target patients who have expectations of additional functional gain.

A limitation of this study is the final follow up rate of ~75 % (*n* = 26) for the number of patients included in the final analysis. Table 1 demonstrates that for the number of recruited and eligible patients (*n* = 49), there is no significant difference in patient characteristics or the baseline assessment of the functional outcomes utilized. The high attrition rate in terms of final analyzed patients means that there is limited generalizability for the results obtained. A further A limitation of the study is that the participants’ additional (not study related) exercise activities (especially relevant for the patients randomized to the SR (control) group) could not be controlled for during the duration of the 6-week intervention period. Another limitation that may have led to the home-based PRT regime not being more effective than standard rehabilitation include the fact that the community physiotherapists who administered the program were also involved in looking after the patients randomised to the SR (control) group. This may have led to some modification of prescription behaviour in dealing with the control group, in terms of adjustment of exercises prescribed (i.e. inclusion of some of the PRT exercises). Additionally, our home-based PRT regime concentrated mainly on training the quadriceps, whilst, most of the studies in the literature involved a variety of exercises which included weight bearing progressive resistance working on hip flexors, extensors, and abductors in a variety of positions [3, 4].

## Conclusions

This study demonstrates that home-based PRT is feasible and well tolerated for patients immediately following THR surgery, and that it is as effective, but not better than standard rehabilitation in improving physical function.

### Ethics statement

This study was approved by the NHS Research Ethics (North West Wales) Committee (Ref 09/WNo01/52) in January 2010. Informed consent was obtained from all participants.

### Consent for publication

Not applicable.

### Availability of data and materials

The CONSORT checklist is provided as Additional file 1. The raw data for this study is available on request from the corresponding author (TO).

## Additional file

Additional file 1: CONSORT 2010 checklist. (DOC 219 kb)

## Abbreviations

6MWT: six minute walk test
ADLs: activities of daily living
ANOVA: analysis of variance
BMI: body mass index
CI: confidence interval
CONSORT: consolidated standards of reporting trials
DEXA: dual energy X-ray absorptiometry
HTVP: high training volume participants
ICC: intra class correlation coefficient
LTVP: low training volume participants
MVC: maximal voluntary contraction
MVCOLQ: Maximal voluntary contraction of operated leg quadriceps
N: Newtons
NFAT: nuclear factor of activated T cells
NHS: national health service
NICE: national institute for health and clinical excellence
NJR: national joint registry
NWORTH: North Wales organisation for randomised trials in health
PRT: progressive resistance training
ROM: range of movement
RT-PCR: reverse transcriptase polymerase chain reaction
SCP: stair climb performance
SD: standard deviation
SR: standard rehabilitation
ST: sit to stand (number of repetitions)
THR: total hip replacement
TO: Tosan Okoro, corresponding author
TUG: timed up and go test (seconds)
